# A missense mutation in the catalytic domain of *O*‐GlcNAc transferase links perturbations in protein *O*‐GlcNAcylation to X‐linked intellectual disability

**DOI:** 10.1002/1873-3468.13640

**Published:** 2019-11-07

**Authors:** Veronica M. Pravata, Mehmet Gundogdu, Sergio G. Bartual, Andrew T. Ferenbach, Marios Stavridis, Katrin Õunap, Sander Pajusalu, Riina Žordania, Monica H. Wojcik, Daan M. F. van Aalten

**Affiliations:** ^1^ Division of Gene Regulation and Expression School of Life Sciences University of Dundee UK; ^2^ Division of Cell and Developmental Biology School of Life Sciences University of Dundee UK; ^3^ Department of Clinical Genetics, United Laboratories Tartu University Hospital Estonia; ^4^ Department of Clinical Genetics Institute of Clinical Medicine University of Tartu Estonia; ^5^ Divisions of Newborn Medicine and Genetics and Genomics Department of Medicine Boston Children’s Hospital Harvard Medical School Boston MA USA; ^6^ Broad Institute of MIT and Harvard Cambridge MA USA

**Keywords:** intellectual disability, neurodevelopment, *O*‐GlcNAc, OGlcNAC transferase, OGT, XLID

## Abstract

X‐linked intellectual disabilities (XLID) are common developmental disorders. The enzyme *O*‐GlcNAc transferase encoded by *OGT*, a recently discovered XLID gene, attaches *O*‐GlcNAc to nuclear and cytoplasmic proteins. As few missense mutations have been described, it is unclear what the aetiology of the patient phenotypes is. Here, we report the discovery of a missense mutation in the catalytic domain of OGT in an XLID patient. X‐ray crystallography reveals that this variant leads to structural rearrangements in the catalytic domain. The mutation reduces *in vitro* OGT activity on substrate peptides/protein. Mouse embryonic stem cells carrying the mutation reveal reduced *O*‐GlcNAcase (OGA) and global *O*‐GlcNAc levels. These data suggest a direct link between changes in the *O*‐GlcNAcome and intellectual disability observed in patients carrying OGT mutations.

## Graphical abstract

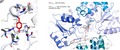

## Abbreviations

**ASD**, autism spectrum disorder

**FP**, fluorescence polarimetry assay

**HCF1**, host cell factor C1

**ID**, intellectual disability

**NGS**, next‐generation sequencing

**OGA**, *O*‐GlcNAcase

**OGT**, O‐β‐N‐acetylglucosamine (GlcNAc) transferase

**TAB1**, TGF‐beta‐activated kinase 1‐binding protein 1

**TIEF**, transferrin isoelectric focusing

**TPRs**, tetratricopeptide repeats

**XLID**, X‐linked intellectual disability

Intellectual disability (ID) is a neurodevelopmental disorder, with a worldwide prevalence of 1% 1, characterised by significant limitations in both intellectual functioning and adaptive behaviour 2. ID is often syndromic, involving not only a limited IQ but also developmental delay, congenital malformations or neurological abnormalities such as autism spectrum disorder (ASD), epilepsy and sensory impairment 3, 4, 5, 6, 7, 8. Although ID can be caused by environmental factors such as maternal alcohol abuse and birth complications 6, 8, 9, genetics plays a crucial role in its aetiology. Mutations, deletions or rearrangements affecting genes involved in development and neuronal function can have severe consequences for proper brain development or cognitive function 10.

To date, approximately 1000 genes have been shown to be involved in ID 11. A disproportionate number (141) of these are located on the X‐chromosome, leading to the coining of the term X‐linked intellectual disability (XLID) 5. In the last few years, next‐generation sequencing (NGS) has led to enormous progress in deciphering monogenic forms of XLID 3, 4, 5, 10. This unbiased NGS approach has accelerated identification of *de novo* mutations in newly described XLID genes, including *OGT,* which encodes *O*‐β‐N‐acetylglucosamine (GlcNAc) transferase (OGT).

OGT catalyses an essential post‐transitional modification, the addition of a single GlcNAc onto serine or threonine of nucleocytoplasmic proteins, using UDP‐GlcNAc as donor substrate 12. OGT is also known to be involved in the processing of the host cell factor C1 (HCF1), the proteolytic products of which are involved in the activation of many genes involved in cell cycle progression 13, 14, 15. The enzyme is divided into a glycosyltransferase catalytic domain and an N‐terminal domain consisting of 13.5 tetratricopeptide repeats (TPRs) that have been shown to contribute to protein substrate binding 16, 17, 18. Together with the hydrolase *O*‐GlcNAcase (OGA), the homeostasis of dynamic protein *O*‐GlcNAcylation is maintained for optimal cellular function 19. Effects of protein *O*‐GlcNAcylation have been linked to protein synthesis 20, stability 21 and turnover 22, 23 and complex processes such as cell cycle progression 24, stress response 25, 26 and transcription 27. *O*‐GlcNAcylation has also been implicated in pathology, including cancer 28, type II diabetes 29, cardiovascular disease 30 and neurodegeneration 31, and it is essential for proper vertebrate development 32, 33, 34, 35. In a range of model systems, *O*‐GlcNAcylation has also been found to be critical for processes in early development. In mice, *Ogt* knockout causes lethality, with mouse embryos dying at the blastocyst stage 36.

*O*‐GlcNAcylation also plays a key role in stem cell biology. Numerous pluripotency factors such as Oct4 and Sox2 have been shown to be *O*‐GlcNAcylated 33, 37. Furthermore, mutations in *OGT* trigger alterations in stem cell differentiation and development, affecting neuronal lineages 37, 38. To date, five *OGT* mutations, all located to the N‐terminal TPR domain, have been reported in patients with XLID 39, 40, 41, 42. These patients display developmental delay, facial dysmorphia, clinodactyly and microcephaly. To date, it is not clear whether the underpinning mechanisms leading to these phenotypes involve changes in the *O*‐GlcNAc proteome.

Here, we report a patient with moderate ID, coarse facial features and a *de novo* mutation located in the catalytic domain of OGT. This mutation induces structural rearrangements in the catalytic domain, leading to reduced levels of protein *O*‐GlcNAcylation *in vitro* and in mESCs, suggesting a direct link between perturbations in protein *O*‐GlcNAcylation and XLID.

## Results and Discussion

### A patient with XLID possesses a missense mutation in the OGT catalytic domain

**Figure 1:**
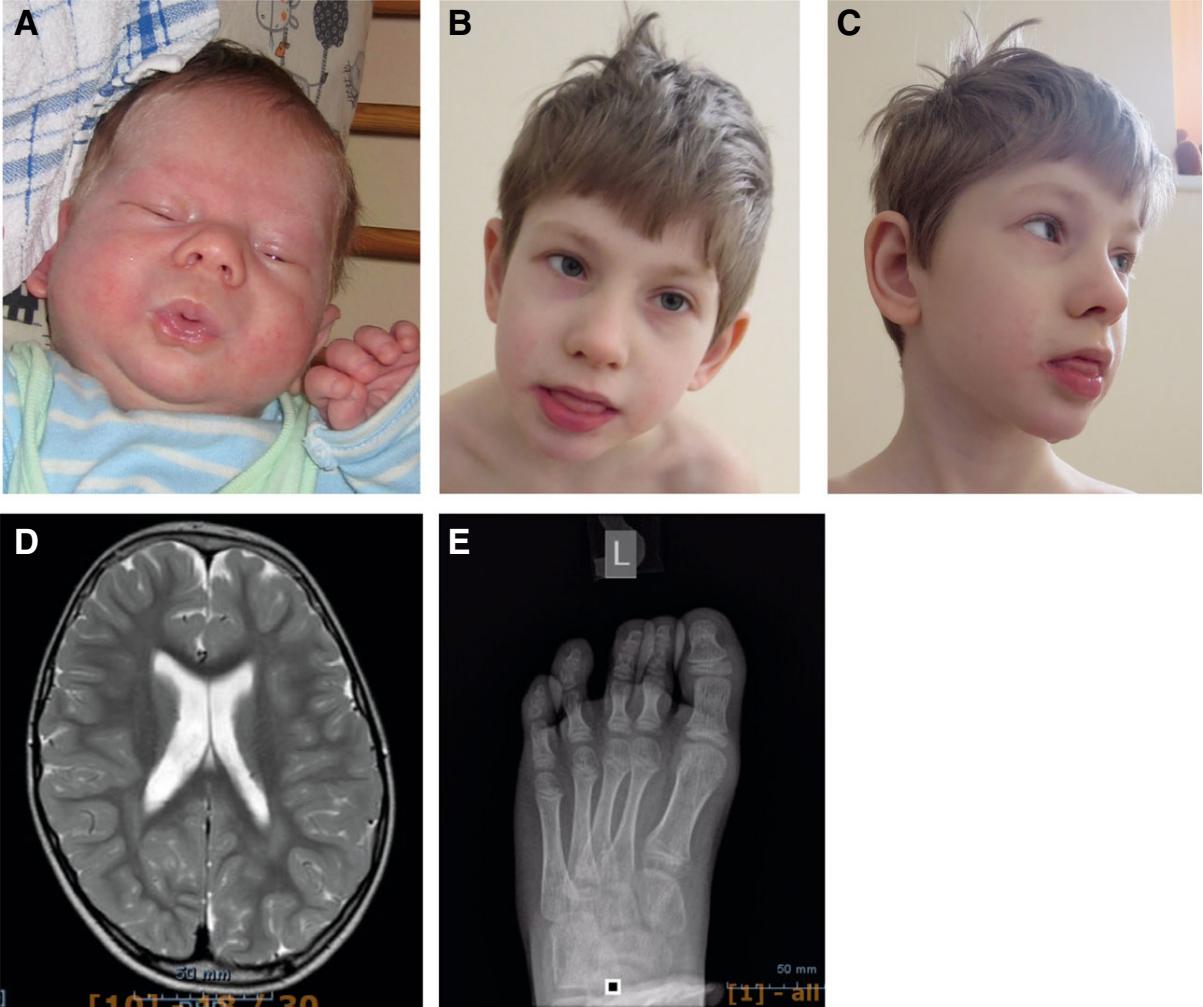
Clinical pictures from patient carrying the N648Y mutation in the catalytic domain of OGT. (A) Facial asymmetry at birth. (B) Facial view at 7 years. (C) Profile view at 7 years. Note coarse facial features, drooling. (D) MRI image showing brain atrophy and mega cisterna magna. (E) X‐ray image of the left foot—cone‐shape epiphyses.

The patient, E.T., was born at term with normal birth weight: 3354 g; length: 50 cm; head circumference: 34.5 cm; and an Apgar score of 9/9. He is the second child of an Estonian nonconsanguineous family. His elder brother is healthy. Facial asymmetry was noticed soon after birth (Fig. 1A). His toenails were also very small and with soft structure at birth. His development was evaluated as normal during the first months of life; he started to hold his head at 3 m and to turn at 6 m of age. Initially, he also had good weight gain, but after some months gastric reflux and gasses became very problematic.

Recurrent otitis started at 4 m, and after narcosis (the shunting of middle ears), his development arrested. His muscular tone became very flaccid. He started to turn at 12 months, to crawl at 14 months, to sit at 22 months and to walk at 3 years of age. First teeth came very late and slowly at 12 months. At the age of 16 month, alimentary vitamin B12 deficiency was diagnosed and treated with intramuscular injections. After that, his development improved significantly. However, at the age of 19 months persistent otitis recurred and his development arrested.

He was subjected to extensive clinical investigation at 19 months due to his developmental delay. He had coarse facial features with open mouth and drooling. He suffered from hypotonia with brisk tendon reflexes and truncal ataxia. There was a suspicion of epilepsy, but Electroencephalogram (EEG) was normal. Brain MRI was normal for that age. On cardiac evaluation, only very mild pericardial effusion was noticed, which did not affect cardiac function. Griffith scale evaluation suggested that his development corresponded to 8 months of age. He had serious problems with obstipation, but no obvious aetiology was detected. Extensive metabolic investigations were performed, which showed normal results (urinary organic acids, glycosaminoglycans, oligosaccharides, sialic acid, creatine/guanidinoacetate and purine/pyrimidines; transferrin isoelectric focusing (TIEF) and acylcarnitine in serum; and neurotransmitters in cerebrospinal fluid). Chromosomal microarray analysis showed no abnormal copy number variations.

At the age of 7 years 8 months, his height was 127 cm (+1 SD), weight 23.4 kg (0 SD) and head circumference 52 cm (−0.5 SD). He is moderately intellectually disabled. He had coarse facial features, convergent strabismus, large ears and intense and constant drooling (Fig. 1B,C). He had no speech and moderately hyperactive behaviour, frequent body jerks, increased sensibility to light and sounds, and fears. In addition, he had hyperelastic connective tissue, which manifested as frequent joint dislocations and scoliosis. He presented mild T2‐3 syndactyly, inverted nipples, abnormally pale skin and body temperature fluctuations in addition to his frequent infections. X‐ray investigation showed cone‐shape epiphyses of T2‐T5 (Fig. 1D), fragmentation of the 1st rib and synostosis of 1‐2 ribs. Brain MRI investigation at 5 years showed brain atrophy and mega cisterna magna (Fig. 1C). Ophthalmological investigation revealed astigmatism and myopia.

Biochemical analyses showed repeatedly increased thyroglobulin (45–48 µg·L^−1^; normal 5–43) and follicle‐stimulating hormone (3.5–3.9 U·L^−1^; normal < 3). All other hormonal analysis and coagulation factors were in normal range. Immunological analysis showed repeatedly low percentage of activated T lymphocytes (1.4–2.1; normal 2.3–7.0 %) and mildly low CD8 T cells % (18%; normal 19–34). TIEF analysis of the serum has been repeatedly performed with normal profile result.

**Figure 2:**
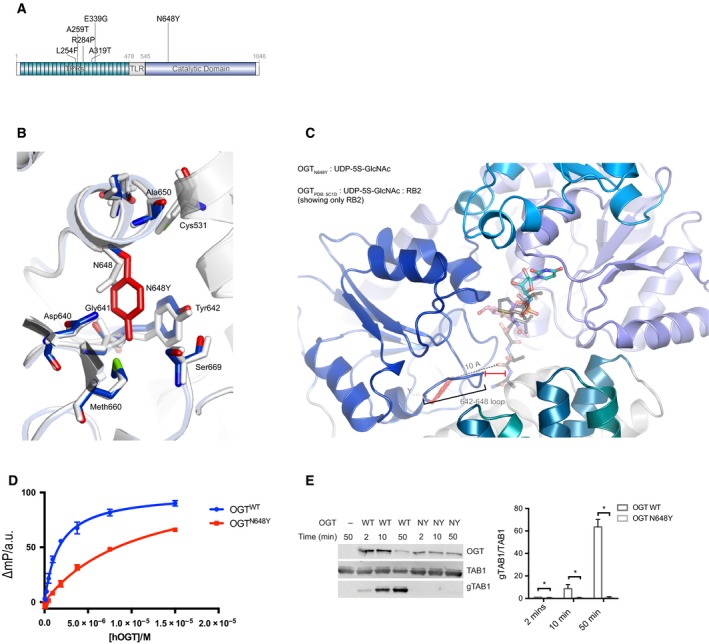
Effects of the N648Y mutation on OGT structure and activity. (A) Schematic diagram of OGT highlighting the TPRs, TPR‐like repeat, the Catalytic domain, the N648Y mutation (bold) and all the previous identified mutations in OGT. TPR, tetratricopeptide repeat domain; TLR, tetratricopeptide repeat‐like domain. (B) OGT superimposed complexes of OGT_WT_ (in light grey; PDB: http://www.rcsb.org/pdb/search/structidSearch.do?structureId=5C1D; 43) and OGT_N648Y_ (in blue; PDB: http://www.rcsb.org/pdb/search/structidSearch.do?structureId=6Q4M) showing the mutated site and the proximal residues. N648Y mutated residue is shown in red. (C) OGT_N648Y_ in complex with superimposed RB2 peptide from OGT_WT_ (PDB: http://www.rcsb.org/pdb/search/structidSearch.do?structureId=5C1D) showing the putative location of the peptide. The loop 642–648 of OGT_N648Y_ is indicated including the distance from the superimposed peptide. (D) FP assay showing the binding of the UDP‐peptide bisubstrate conjugate to OGT_WT_ and OGT ^N648Y^. (E) Immunoblots showing OGT glycosyltransferase activity against TAB1 and gTAB1. Quantification of gTAB1 normalised to TAB1 signal. *N* = 3, mean ± SD. Multiple *t*‐test using the Holm–Sidak method. * corresponds to *P* = 0.021 (2 min), *P* = 0.017 (5 min) and *P* = 0.008 (50 min) TAB1, TAK1‐binding protein antibody; gTAB1, glycosylated TAB1 antibody.

Trio whole‐genome sequencing was performed. After variant filtration for *de novo*, recessive or X‐linked variants with allele frequencies below 1%, we identified a missense variant in the *OGT* gene ChrX (GRCh38): g.71561865A>T; NM_181672.2: c.1942A>T p.(Asn648Tyr). Both parents and sibling were healthy and did not carry this mutation. Unlike the other OGT XLID mutations identified to date, this mutation maps to the catalytic domain of OGT (Fig. 2A).

### The N648Y mutation leads to structural changes in the catalytic domain

Asn648 is highly conserved in OGT from *C. elegans* to *H. sapiens* (Fig. S1). Inspection of the human OGT crystal structure reveals that Asn648 maps to the interface of the OGT TPRs with the catalytic domain. The Asn648 side chain forms van der Waals interactions with that of Tyr642, while the loop between these two interacting residues (hereafter 642–648 loop) forms part of the composite OGT acceptor substrate binding cleft. It is thus possible that this mutation could affect the TPR‐catalytic domain interface and lead to changes in the stability of the protein. We first analysed the effect of the mutation on the folding stability using differential scanning fluorimetry. Using an *E. coli* expression system, we purified a recombinant truncated form of OGT version containing the catalytic domain and 3.5 TPR repeats, for both wild‐type (OGT^WT^) and mutant (OGT^N648Y^) OGT. Sigmoidal temperature‐induced unfolding curves were obtained for both OGT^WT^ and OGT^N648Y^ with inflection points (*T_m_*) of 44 °C, indicative of identical unfolding temperatures (Fig. S2). Given that the mutation has the potential to alter the 642–648 loop, which forms an important part of the catalytic pocket, we next investigated possible structural changes by X‐ray crystallography. Recombinant OGT^N648Y^ was crystallised in the presence of the donor substrate analogue UDP‐5S‐GlcNAc and an acceptor peptide derived from the well‐characterised OGT substrate TGF‐beta‐activated kinase 1‐binding protein 1 (TAB1). Diffraction data were collected to 2.2 Å, and initial refinement started from the published OGT^WT^ ternary structure 43. Mutation of Asn648 to the bulkier tyrosine appears to be accommodated without disruption of the domain organisation observed in the wild‐type structure (RMSD of 0.4 Å on all backbone Cα atoms) (Fig. 2B). However, the aromatic Tyr648 side chain forms a π‐π stacking interaction with Tyr642 causing positional shifts of up to 0.7 Å in the 642–648 loop. Strikingly, despite the presence of an acceptor peptide in the crystallisation condition, no corresponding electron density was observed in the OGT active site. This could be because the changes in 642–648 loop caused by the mutation affect the ability of OGT to bind to acceptor substrates (Fig. 2C). To explore this possibility, we used a recently established fluorescence polarimetry assay (FP) where increasing concentrations of OGT are titrated against a fixed concentration of a fluorescent probe that incorporated elements of both peptide and nucleotide substrates 44. The direct binding affinity of the probe to OGT^WT^ yielded a *Kd *value of 1.6 µm as previously reported, whereas the *Kd* value for the OGT^N648Y^ was 7 times higher (11 µm), suggesting reduced substrate binding in agreement with the structural observations (Fig. 2D). In summary, the N648Y mutation may lead to changes in the OGT acceptor substrate binding cleft and affect substrate binding.

### OGT^N648Y^ does not glycosylate the model acceptor substrate TAB1

Given the absence of effects on stability and the apparent effect of the N648Y mutation in OGT substrate binding, it is possible that catalytic activity is affected. To investigate this, we used an enzyme assay where we incubated OGT^WT^ or OGT^N648Y^ with the well‐characterised substrate TAB1. This substrate is efficiently monoglycosylated on Ser395 by wild‐type OGT 45. Western blotting analysis using a specific antibody which binds the *O*‐GlcNAcylated form of TAB1 45 showed that the OGT^N648Y^ variant was unable to glycosylate the substrate (Fig. 2E), in contrast to the efficient glycosylation observed by OGT^WT^. Thus, OGT^N648Y^ does not glycosylate the model acceptor substrate TAB1*.*

### The N648Y mutation leads to hypoglycosylation in mES cells

**Figure 3:**
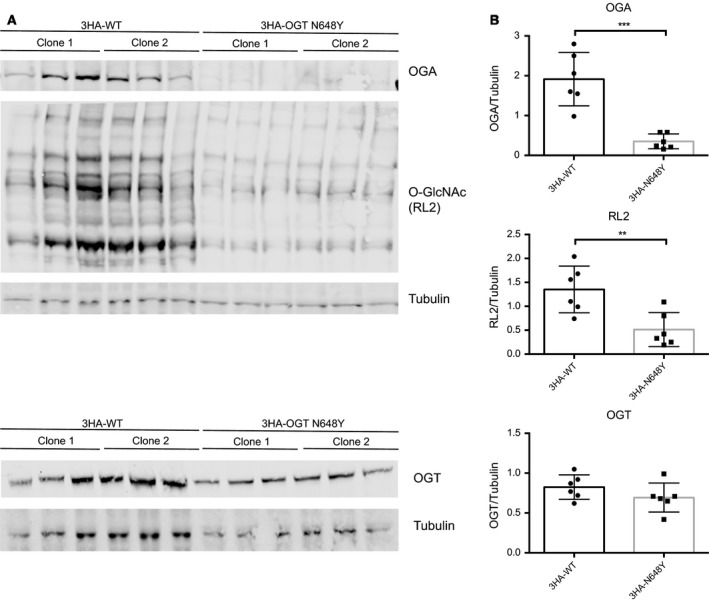
The N648Y mutation leads to reduced protein *O*‐GlcNAcylation in 3HA‐N648Y mES cells. (A) Immunoblots showing OGA, protein *O*‐GlcNAcylation (RL2) and OGT levels in 3HA‐WT and 3HA‐N648Y undifferentiated mES cells. (B) Quantification of western blotting of OGA, protein *O*‐GlcNAcylation (RL2) and OGT levels normalised to tubulin signal. *n* = 3, mean ± SD. Unpaired *t*‐test. ** corresponds to *P* = 0.0068 (RL2); *** corresponds to *P* = 0.0003 (OGA).

Maintenance of *O*‐GlcNAc homeostasis is essential for optimal cellular function, and perturbations in protein *O*‐GlcNAcylation have been implicated in pathogenesis. We next investigated the effects of reduced OGT^N648Y^ activity on *O*‐GlcNAc homeostasis and levels of OGA/OGT. We used CRISPR/Cas9 genome editing to introduce the N648Y mutation in the endogenous *ogt* locus carrying an additional N‐terminal triple HA (3HA) tag (as described in detail in Materials and methods). Two independent clones each were obtained for mESCs carrying 3HA‐tagged wild‐type and 3HA‐tagged mutant OGT, as verified by diagnostic restriction digest and sequencing. Western blotting analyses of 3HA‐N648Y mESC cells were carried out to analyse OGT, OGA and protein *O*‐GlcNAcylation levels (Fig. 3A). In agreement with loss of *in vitro* activity towards the TAB1 substrate (Fig. 2E) and the potential effect of the loop 642–648 on OGT substrate binding (Fig. 2D), this experiment revealed significantly reduced *O*‐GlcNAc levels in 3HA‐N648Y mESCs compared to the controls. This is evidence for a possible link between perturbation in protein *O*‐GlcNAcylation and XLID. No reduction in OGT protein levels was detected. However, OGA levels appeared reduced in 3HA‐N648Y mESCs (Fig. 3B). This hints at the existence of a compensatory mechanism that allows cells to partially maintain *O*‐GlcNAc homeostasis by reducing OGA levels—as also observed in some of the other OGT XLID mutations in the TPR domain 39, 46. Thus, the N648Y mutation leads to hypoglycosylation in mES cells.

## Concluding remarks

Protein *O*‐GlcNAcylation has emerged as an essential PTM involved in disease and more recently in neuronal function and development 19, 32, 33, 41. Recent NGS studies have led to the identification of mutations in *OGT* that give rise to XLID, suggesting a direct link between *O*‐GlcNAc and regulation of brain development. However, experimental evidence for a link between significant loss of OGT activity and changes in the O‐GlcNAc proteome has remained elusive, presumably because of the location of these mutations in the N‐terminal TPR substrate binding domain of OGT. Here, we report a patient with XLID attributable to an *OGT* missense mutation (Asp648Tyr), which maps to the OGT catalytic domain (Fig. 2A). In agreement with the five previously reported patients, the patient shows reduced IQ, limited speech, developmental delay, facial dysmorphia and hypotonia.

We initially delineated the effects of the mutation on the stability, structure and activity of the enzyme using *in vitro* approaches. Unlike the previously reported OGT XLID mutations, we were not able to detect changes in folding stability induced by the mutation (Fig. S2). Using X‐ray crystallography, we revealed that the mutation did not induce large global conformational changes. However, the observed local conformational changes in the OGT acceptor substrate binding cleft could affect activity. Indeed, using *in vitro* analyses, the mutant led to significant reduction in the binding affinity of a bisubstrate fluorescence polarimetry probe and defects in the glycosylation of TAB1, suggesting a direct effect of the mutation on enzymatic activity (Fig. 2D,E).

To extend our investigations, we introduced the patient mutation into a male mouse embryonic stem cell line. In previous studies, examination of the steady‐state global OGT, OGA and *O*‐GlcNAc levels in different cell lines showed no significant changes in protein *O*‐GlcNAc and OGT, while OGA levels appeared reduced in most of the analysed cell lines, suggesting a compensatory mechanism between OGT and OGA to maintain *O*‐GlcNAc homeostasis (Fig. 3). To our surprise, the 3HA‐N648Y mESC lines showed not only reduced levels of OGA, but also reduced protein *O*‐GlcNAcylation levels. This suggests that perturbations in *O*‐GlcNAc cycling may contribute to the patient ID phenotype. This now enables the search for specific proteins and *O*‐GlcNAc sites, whose altered *O*‐GlcNAcylation levels can be linked to cellular processes that could be causative for the observed phenotypes.

There is currently only a very limited number (6) of XLID patients identified that carry different mutations in *OGT*. It is thus not yet feasible to consider possible treatments. An interesting clue to a possible treatment is the observed changes in OGA levels. This is a mechanism that may compensate for loss in OGT activity or protein levels. This would lead to the hypothesis that (further) inhibition of OGA is a possible route towards re‐establishing full *O*‐GlcNAc homeostasis and possible alleviation of the patient phenotypes. Another route would be to increase cellular production of UDP‐GlcNAc, which has been shown to directly lead to increased levels of protein *O*‐GlcNAcylation. It has been demonstrated that this is possible to achieve by feeding cells with the UDP‐GlcNAc precursor glucosamine, which enters the hexosamine biosynthetic pathway as glucosamine‐6‐phosphate and leads to increased UDP‐GlcNAc levels 47, 48, 49, 50. Glucosamine, in the form of glucosamine sulfate, is a food supplement frequently taken by patients suffering from osteoarthritis, albeit with limited clinical evidence for significant benefits 51, 52. In an initial experiment, at the age of 8 years of our patient we started the treatment with glucosamine sulfate (400 mg three times a day). This treatment has been performed over a period of 13 months without any adverse effects (unpublished data). Parents have noticed that the patient is more active; he has more energy and has increased plasticity in movements. He started to use syllables, and teeth development progressed. However, there are no changes in clinical biochemical analyses, and in the absence of a proper randomised trial, it is not possible to attribute these improvements to the treatments. Nevertheless, this is an approach that could be evaluated once sufficient patient numbers are available.

## Materials and methods

### Whole‐genome sequencing

Trio whole‐genome sequencing and data processing were performed by Genomics Platform at the Broad Institute of Harvard and MIT (Broad Institute, Cambridge, MA, USA). The variant filtration for *de novo*, recessive or X‐linked variants with allele frequencies below 1% in ExAC and gnomAD databases revealed three high‐quality variants, all appearing hemizygous on X‐chromosome in genes DLG3, OGT and KIAA1210. Out of them, the OGT variant ChrX(GRCh38): g.71561865A>T; NM_181672.2: c.1942A>T p.(Asn648Tyr) was the only variant with no hemizygous carriers in ExAC, gnomAD and in‐house database, and thus considered as the most probable candidate after variant filtration. The variant appeared as de novo in protein domain: *O*‐GlcNAc transferase, C‐terminal. The variant was validated by Sanger sequencing. The mutated p.Asn648 amino acid is highly conserved from man to *C. elegans*, and multiple *in silico* pathogenicity predicting algorithms indicated a damaging effect. Pathogenicity classified by the American College of Medical Genetics variant interpretation guidelines is likely pathogenic (absent from population databases, multiple computational evidence and *de novo*).

### Protein expression and purification

Truncated (323–1044) OGT constructs were expressed in *E. coli* BL21(DE3) RIPL cells as N‐terminal GST fusion protein, as described previously 43. Briefly, transformed *E. coli* cells were grown in LB broth at 37 °C with agitation until OD_600_ reached 0.8, at which point the temperature was lowered to 18 °C and expression was induced by addition of 100 µm IPTG. Cells were lysed using French Press in base buffer (0.1 m Tris/HCl, pH 7.5, 0.15 m NaCl, 0.5 mm TCEP) supplemented with 25 mm imidazole, 0.1 mg·mL^−1^ DNase I and protease inhibitor cocktail (1 mm benzamidine, 0.2 mm PMSF, 5 mm leupeptin). Then, recombinant proteins were affinity‐purified following manufacturers’ guidelines. After cleavage of affinity tags, dialysed protein was loaded onto 5 mL HiTrap Q Sepharose FF anion exchange resin (GE Healthcare) and eluted with a linear gradient up to 60% of buffer B (0.1 m Tris/HCl, pH 8.5, 500 mm NaCl). Peak fractions were pooled, concentrated and further purified via size exclusion chromatography using a 300 mL Superdex™ 200 column (GE Healthcare) equilibrated with base buffer. The peak fractions were concentrated to 10 mg·mL^−1^. For crystallisation, truncated mutant OGT was used fresh at 7 mg·mL^−1^ concentration. For all other purposes, proteins were concentrated to 10 mg·mL^−1^, mixed 1 : 1 with 50% glycerol, snap‐frozen and stored at −80 °C until use.

### Structure solution

Crystallisation of truncated OGT_N648Y_ (residues 323–1044) was performed as described previously 43. Briefly, experiments were performed at 22 ºC using 24‐well hanging drop crystallisation plates, by combining 1 µL drops containing 7 mg·mL^−1^ OGT_N648Y_ (in base buffer), 3 mm UDP‐5S‐GlcNAc and 3 mm acceptor peptide derived from TAB1 sequence (PVSVPY**S**SAQSTS) with 2 µL of reservoir solution (1.45 m K_2_HPO_4_, 8 mm EDTA and 1% xylitol). Large, clear‐faced crystals appeared overnight. Prior to diffraction experiments, individual crystals were cryoprotected in reservoir solution supplemented with 3.5 m maleic acid, 3 mm UDP‐5S‐GlcNAc and 3 mm acceptor peptide and flash‐frozen in liquid nitrogen. Diffraction data were collected at the European Synchrotron Radiation Facility beamline ID23. Data were processed with XDS 53 and scaled to 2.2 Å using Scala 54. The structure was solved by molecular replacement using the structure for OGT_WT_ (PDB: http://www.rcsb.org/pdb/search/structidSearch.do?structureId=5C1D; 43) as the search model. The resulting model was manually refined using Coot 54 and REFMAC 55, respectively. Although the mutant protein was crystallised in the presence of donor and acceptor, there was no evidence for the latter in the electron density maps and was therefore excluded from the model. Coordinates and structure factors were deposited in the PDB (PDB: http://www.rcsb.org/pdb/search/structidSearch.do?structureId=6Q4M). Scaling and model building statistics can be seen in Table 1.

**Table 1:** X‐ray diffraction data processing and refinement statistics of the OGT_N648Y_ ternary complex. Numbers in brackets show represent the highest resolution bin.

	OGT_N648Y_
Space group	*F*222
Cell dimensions	
α, β, γ (°)	137.3, 150.7, 199.5
Resolution (Å)	45.92–2.27 (2.20–2.27)
*R*_sym_ or *R*_merge_	0.08 (0.87)
*I*/ σ*I*	12.0 (2.0)
Completeness (%)	100 (99)
Redundancy	6.7 (6.7)
No. of reflections	52 373 (819)
*R*_work_/*R*_free_	0.17/0.20
RMSD	
Bond lengths (Å)	0.011
Bond angles (°)	1.8

### Fluorescence polarimetry assay

To evaluate the expected binding differences between the OGT^WT^ and the OGT^N648Y^, we titrated a previously published fluorescently labelled UDP‐peptide bisubstrate conjugate 56 with increasing concentrations of either OGT^WT^ or the OGT^N648Y^ in 0.1 m Tris/HCl pH 7.5, 0.15 m NaCl, 0.5 mm TCEP and 5% DMSO. Each sample, containing 25 μL, was incubated in the dark for 30 min before the read‐out with a PHERAstar plate reader (BMG LABTECH). Subsequent data analysis was performed using graphpad prism 6 as outlined previously 56.

### Activity assays

*O*‐GlcNAcylation assays were performed on TAB1 protein (residues 7–420). TAB1 (1 µm) was combined with full‐length OGT_WT_ or OGT_N648Y_ (0.1 µm) in the presence of 100 µm UDP‐GlcNAc. The reaction mixtures were incubated at 37 ºC for 2–50 min and subsequently stopped by addition of LDS loading buffer (4x, Thermo Fisher Scientific). Proteins were resolved by SDS/PAGE (4–12% acrylamide [Life Technologies]) and transferred onto nitrocellulose membranes (GE Healthcare). After antibody treatment, progress of the reaction was visualised using LI‐COR Odyssey Scanner and associated quantification software.

### Mouse ES Cell culture

mESC E14‐TG2a.IV AW2 line was acquired from the MRC Centre for Regenerative Medicine, Institute for Stem Cell Research, University of Edinburgh 57. mESCs were cultured in an undifferentiated state in 0.1% gelatine [w/v]‐coated plates in GMEM BHK‐21 (Gibco) supplemented with 10% FBS [v/v] (Gibco), 0.1 mm MEM nonessential amino acids (Gibco), 1 mm sodium pyruvate (Gibco), 0.1 mm 2‐mercaptoethanol (Gibco) and 100 units·mL^−1^ LIF (produced in house) at 37 ºC in 5% CO_2_.

### Generation of 3HA‐tag N567K mES cell line

For the generation of the 3HA mES cell line, we transfected wild‐type male E14‐TG2a.IV AW2 mES cells with pBABED puro U6 and pX335 (Cas9 D10A) vectors containing the gRNA sequences selected using WTSI Genome Editing 58. Silent mutations were designed in addition to the intended mutations, removing *Pst*I and *Bfm*I restriction sites (Fig. S2). A gene block containing these changes was obtained from IDT (International DNA technologies). The gene block was introduced into the cloned 2 kb region by restriction‐free cloning 59 and then confirmed by DNA sequencing 59. For transfection, 2 × 10^5^ cells were seeded onto gelatine‐coated 24‐well plates and transfected using 0.33 µg of each vector and Lipofectamine 3000 according to manufacturer’s instructions. After 24 h, media was replaced and puromycin (3 µg·mL^−1^) was added to the cells for selection until control untransfected cells were all dead (48 h). Then, the cells were replated into 10‐cm plates for recovery. After that, single‐cell selection was carried out using limited dilution according to 0.3 cell per well in 96‐well plates. For confirmation of mutations, diagnostic restriction digests and genomic DNA sequencing analysis were carried out. For the restriction fragment length polymorphism assay, 3HADiag_F and 3HADiag_R primers were used to amplify by PCR the mutated site and the silent mutation which eliminates a *Pst*I restriction site (Table S1). The size of the PCR product (546 bp vs 450 bp for wild‐type) was used to screen for successful integration. To further confirm, the PCR product was then digested using *Pst*I or *Bfm*I. Clones negative for the restriction enzyme assay were then sequenced to confirm the presence of the modification.

For generation of the 3HA‐OGT^N648Y^ mESCs, we transfected the previously generated 3HA mESCs with pBABED puro U6 and pX335 (Cas9 D10A) vectors containing the gRNA sequences using the same procedure we previously used for the insertion of the 3HA‐tag (Fig. S3). Restriction enzymes and genomic DNA sequencing analysis were used for mutation identification. For the restriction fragment length polymorphism assay, MouseEstDiag_F and MouseEstDiag_R primers were used to amplify by PCR the mutated site and the silent mutation which eliminates an *Eco130I* restriction site (Table S2). The size of the bands following digestion (167 bp and 433 bp for wild‐type versus 600 bp for mutant) was used to screen for successful integration. Clones negative for cutting in the restriction enzyme assay were then sequenced to confirm the presence of the modification.

### Western Blot

Cells were harvested in lysis buffer containing 150 mm NaCl, 1% Nonidet P‐40, 0.5% sodium deoxycholate, 0.1% sodium dodecyl sulfate, 25 mm Tris/HCl (pH 7.4), 1 mm sodium orthovanadate, 50 mm sodium fluoride and 5 mm sodium pyrophosphate. 10–20 µg of cell lysate was loaded in NuPAGE 3‐8% Tris/acetate gels (Invitrogen) and transferred to nitrocellulose membranes using wet transfer system. Membranes were blocked with TBS‐T 5% BSA buffer [w/v]. Primary antibodies used were OGT (F‐12, Santa Cruz Biotechnology 1 : 5000), OGA (Sigma 1 : 500) and RL2 (Thermo Fisher 1 : 1000).

## Author contributions

VMP, MG and DMFvA conceived the study; VMP, ATF, SGB and MG performed experiments; VMP and MG analysed data; VMP, MPS and DMFvA interpreted the data and wrote the manuscript with input from all authors; KÕ and RŽ collected clinical data and diagnostic samples; and SP, KÕ and MHW analysed genomic data.

## Supporting information


**Fig. S1.** Sequence alignment shows the highly conserved Asparagine 648 from *H. sapiens* to *C. elegans*.
**Fig. S2.** Effects of the N648Y mutation on unfolding temperature.
**Fig. S3.** Gene‐editing of mouse ES cells to introduce 3HA‐tag into the endogenous OGT gene.
**Fig. S4.** Gene‐editing of mouse ES cells to introduce N648Y mutation into endogenous OGT gene.
**Table S1.** Primers and geneblock used for introducing 3HA‐tag to OGT gene and genotyping candidate 3HA‐OGT^WT^ mES cell line.
**Table S2.** Primers and geneblock used for introducing N648Ymutation to OGT gene and genotyping candidate 3HA‐OGT^N648Y^ mES cell line.
