# Supplementary material for: A missense mutation in the catalytic domain of O‐GlcNAc transferase links perturbations in protein O‐GlcNAcylation to X‐linked intellectual disability
Source: FEBS Lett. 2019 Nov 7;594(4):717–27. doi: 10.1002/1873-3468.13640 (PMC7042088; doi:10.1002/1873-3468.13640)
Supplement: Supplementary file 1 — Fig. S1. Sequence alignment shows the highly conserved Asparagine 648 from H. sapiens to C. elegans. Fig. S2. Effects of the N648Y mutation on unfolding temperature. Fig. S3. Gene‐editing of mouse ES cells to introduce 3HA‐tag into the endogenous OGT gene. Fig. S4. Gene‐editing of mouse ES cells to introduce N648Y mutation into endogenous OGT gene. Table S1. Primers and geneblock used for introducing 3HA‐tag to OGT gene and genotyping candidate 3HA‐OGTWT mES cell line. Table S2. Primers and geneblock used for introducing N648Ymutation to OGT gene and genotyping candidate 3HA‐OGTN648Y mES cell line. [file FEB2-594-717-s001.docx]

# **Supporting information**


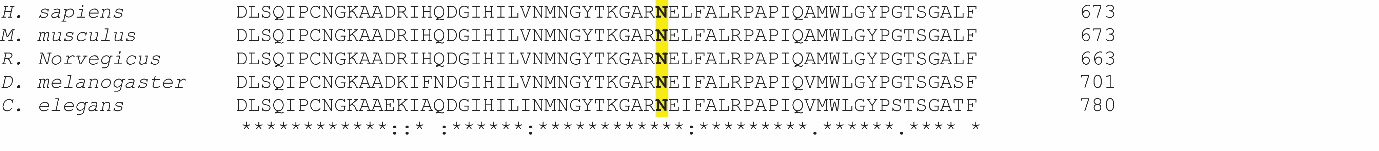


**Supplementary figure S1: Sequence alignment shows the highly conserved Asparagine 648 from *H. sapiens* to *C. elegans*.**

Sequence alignment analysis showing that the Asp648 is highly conserved across species. Clustal Omega was used to generate alignments between sequences (Madeira et al., 2019).

**Supplementary figure S2: Effects of the N648Y mutation on unfolding temperature.**

Thermal denaturation curve showing fraction of unfolded OGT_WT_ and OGT_N648Y_ as a function of temperature as measured with differential scanning fluorimetry. Data averaged from 12 replicates were fitted to a Boltzmann sigmoidal curve equation, with error bars representing SD.

**Supplementary Figure S3: Gene-editing of mouse ES cells to introduce 3HA-tag into the endogenous OGT gene.**

Genomic DNA sequence of mouse wild type and 3HA OGT gene and protein product is shown (highlighted in yellow). *Bfm*I and *Pst*I restriction sites are lost in 3HA cells (silent mutations highlighted in green)

**Supplementary Figure S4: Gene-editing of mouse ES cells to introduce N648Y mutation into endogenous OGT gene.**

Genomic DNA sequence of mouse wild type and N648Y OGT gene and translated protein product is shown. Modification highlighted in orange is responsible for the N648Y mutation, nucleotides highlighted in green are silent wobble sites. *Eco*130I restriction site is lost in N567K cells.

| **Guide RNA** | L3HA_F | CACCGtaattccattgtgtattgtt |
| --- | --- | --- |
| **Guide RNA** | L3HA_R | AAACaacaatacacaatggaattaC |
| **Guide RNA** | 3HAR_F | CACCGagcgactttatctgcagatg |
| **Guide RNA** | 3HAR_L | AAACcatctgcagataaagtcgctC |
| **Genomic DNA** | MmOGT_HA_F | aaaGGATCCaggcaggaggattgccttaagttttagg |
| **Genomic DNA** | MmOGT_HA_R | aaaGCGGCCGCgaaagaaatgggaagaacaggactcac |
| **Sequencing** | MmOGT_HA_seq1 | Gaaagtgtcccacagacatg |
| **Sequencing** | MmOGT_HA_seq2 | gaatctcttagagttttgcagc |
| **Mutagenic GB*** | MmOGT_HA_GB | ctgatttgtaaatttggttctctttgtttttaccacctagcctgaagaaaattcgtggcaaagtctggaaacagagaatatctagccctctgttcaacacAaaGcaGtaTacCatggaattagagcgactGtaCctCcaAatgtgggagcattatgcagctggcaacaaacctgaccacatgattaagcctgttgaagtcaccgagtcagccTCTAGATACCCATACGATGTTCCTGACTATGCGGGCTATCCCTATGACGTCCCGGACTATGCAGGATCCTATCCATATGACGTTCCAGATTACGCTtgaataaagactgcgcacaggagaattgccctatacctgagcctcaaccttctgggggaagggaactagataacatgctttgtgtgtatctgtgtagttctgtgttgcag |
| **Mutagenesis** | 3xHA_fwd | ctgatttgtaaatttggttctctttg |
| **Mutagenesis** | 3xHA_rev | ctgcaacacagaactacacagatac |
| **Genotyping** | 3HAdiag_F | CTGGCCTGGGTCATTCCTCATTAATC |
| **Genotyping** | 3HAdiag_R | CAATTCTCCTGTGCGCAGTCTTTATTC |

**Supplementary Table S1: Primers and geneblock used for introducing 3HA-tag to OGT gene and genotyping candidate 3HA-OGT^WT^ mES cell line.**

* - upper case letters denote introduced changes in Geneblocks (GB).

| **Guide RNA** | MmEsLEF_F | CACC gagctcattccgagcaccct |
| --- | --- | --- |
| **Guide RNA** | MmEsLEF_R | AAAC agggtgctcggaatgagctc |
| **Guide RNA** | MmEsRIG_F | CACCG tcttaggccagctcctattc |
| **Guide RNA** | MmEsRIG_R | AAAC gaataggagctggcctaagaC |
| **Genomic DNA** | V3_MmEsto_BamHI_F | aaaGGATCC  gagtctgttatagtcctctgtttatacc |
| **Genomic DNA** | V3_MmEsto_NotI_R | aaaGCGGCCGC ctgcaatcgtattcatgggaataacgg |
| **Sequencing** | MmEsto_seq1 | Ttgctgggaattgaactcaag |
| **Sequencing** | MmEsto_seq2 | Atcagtggaaacaggcatttc |
| **Mutagenic GB*** | MmOGT_HA_GB | attccttgtaatggaaaagcagccgaccgcatccaccaagatggaattcacatccttgtgaatatgaatgggtatacAaaAggCgcCcgCTatgagctctttgctcttaggccTgcCccCatCcaggtaaaagaacaatcacttacaatgtctattggtctgaaaagatagtgggttttgggttttctccatctggctaactgctct |
| **Mutagenesis** | MmEstoPatch_F | gatggaattCacatccttgtgaatatg |
| **Mutagenesis** | MmEstoPatch_R | gaccaatagacattgtaagtgattg |
| **Genotyping** | MmESTO_DIG_F | tacagatggttgtgagccaccacgtg |
| **Genotyping** | MmESTO_DIG_R | cacctaaaaatctatcctcgtccattcc |

**Supplementary Table S2: Primers and geneblock used for introducing N648Ymutation to OGT gene and genotyping candidate 3HA-OGT^N648Y^ mES cell line.**

* - upper case letters denote introduced changes in Geneblocks (GB).
